# Hysteresis in Perovskite Devices: Understanding the Abrupt Resistive Switching Mechanism

**DOI:** 10.1021/acsenergylett.5c01556

**Published:** 2025-07-24

**Authors:** Agustin O. Alvarez, Jeroen J. de Boer, Lars Sonneveld, Yorick Bleiji, Esther Alarcón-Lladó, Bruno Ehrler

**Affiliations:** † 55952 AMOLF, Science Park 104, 1098 XG Amsterdam, The Netherlands; ‡ van ‘t Hoff Institute for Molecular Sciences, Universiteit van Amsterdam, 1012 WX Amsterdam, The Netherlands

## Abstract

Halide perovskite devices exhibit diverse current–voltage hysteresis behaviors, driven by distinct mechanisms that can enhance or hinder performance, making their understanding crucial. Among these, abrupt switching is particularly relevant for memristive operation and reverse-bias breakdown in solar cells. In this work, we identify four distinct hysteresis responses: capacitive, inductive, hysteresis-free, and abrupt switching. All four behaviors are clearly observed via cyclic voltammetry in a simple perovskite device with silver contacts. Real-time photoluminescence microscopy shows that continuous bias and illumination progressively modify the perovskite–electrode interface, transforming inductive into hysteresis-free behavior and supporting its interfacial origin. Further stress leads to filament formation, with abrupt switching occurring only when a filament bridges the electrodes, forming a reversible short circuit. This switching arises from dynamic contact at the filament-electrode interface. Conductive AFM and electron microscopy reveal that the filaments are highly conductive and composed of metallic silver. Transient and impedance measurements effectively differentiate the hysteresis modes. Similar responses are found in gold-contacted devices, though abrupt switching is restricted to nanometer-scale gaps between the electrodes, suggesting the formation of smaller, less stable filaments due to the lower reactivity of gold. These findings provide valuable insights for advancing switching and understanding hysteresis in perovskite-based devices.

## Graphical abstract

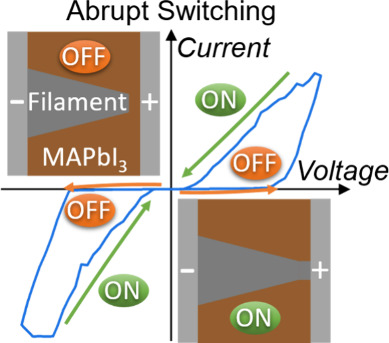

Metal halide perovskites have emerged as a promising class of semiconductors for diverse optoelectronic applications, including solar cells, LEDs, X-ray detectors, and memristors.^1–5^ A notable characteristic of perovskite-based devices is their tendency to exhibit dynamic resistance changes over extended time scales in response to conditions such as applied voltage or illumination. These resistance dynamics can be observed as hysteresis through cyclic voltammetry measurements. Different types of hysteresis have been observed, and they have been associated with reduced performance and stability in perovskite solar cells^6^ and other devices like X-ray detectors,^7–9^ while being exploited for the development of perovskite-based memristors.^10,11^ Despite the rapid advancements in the performance of halide perovskite-based devices, key questions remain regarding the mechanisms behind the hysteresis, which arises from the interplay of electronic and ionic processes.^12–15^ Understanding and controlling these processes is crucial for advancing device applications.

The field of memristors is an exciting and rapidly growing research area, driven mainly by its potential for memory storage and neuromorphic computing.^16–18^ Descriptions of the mechanisms of resistance dynamics in halide perovskite memristors and related definitions in the memristor field can vary across different studies and sometimes conflict.^19^ Here we clarify the different operational mechanisms behind the resistance dynamics.

In this context, we present the definitions used in this work. A memristor (short for memory resistor) refers to any two-electrode system whose resistance depends on previously applied voltage or current.^20^ Memristors can be broadly classified as either volatile or nonvolatile, depending on the stability of the resistance state once power is removed. Volatile memristors reset to their original state upon power loss, while nonvolatile types retain their resistance state,^21^ both of which are promising for different applications.^22,23^ Cyclic voltammetry (CV) is the most standard, straightforward, and rapid technique for characterizing resistance dynamics in optoelectronic devices. Hysteresis is observed as the differences between the forward (increasing voltage) and reverse (decreasing voltage) voltage scans. In perovskite solar cells, this hysteresis, typically measured at scan rates around 1 V/s, is dominated by ionic processes rather than faster purely electronic effects.^6,24^ *Capacitive* (or *Normal*) *hysteresis*, where the current is higher during the forward scan, is related to a capacitive behavior and associated with ionic accumulation at the interfaces.^12,25^ *Inductive* (or *Inverted*) *hysteresis*, where the current is higher during the reverse scan, is related to a negatively capacitive (or inductive) behavior and associated with a recombination process driven by ionic movement.^26^ The current in these definitions is considered to be positive when flowing through the device from the positive to the negative electrode. For solar cells, the opposite definition is often employed. Both inductive and capacitive behaviors have been used to create memristors from perovskite-based devices.^27,28^ In both cases, these memristors are volatile, returning to their initial state once power is removed.

Another resistance change behavior, known as abrupt switching, is commonly used to create both volatile and nonvolatile memristors.^29,30^ In CV measurements, it appears as a sudden increase in current due to a rapid decrease in resistance. For a device to function as a memristor, it must be able to return to its initial high-resistance state, either spontaneously as the voltage decreases (volatile memristor) or by applying a reverse voltage (nonvolatile memristor), both promising for computing applications.^31^ The resulting CV exhibits higher current in the reverse bias than in the forward bias, resembling inductive hysteresis, which in some cases may cause misinterpretation.

Proposed mechanisms for the *abrupt switching* in halide perovskite memristors include filament formation, ionic migration, and interfacial mechanisms.^10,32–34^ Different terms have been used for this behavior, including “threshold switching”^35^ or “filamentary switching”.^36^ Filaments have been directly observed in halide perovskite memristors when silver contacts are used.^36,37^ However, the mechanism behind the abrupt resistance switching, correlating the current increase with the filament growth, and the distinction with the capacitive and inductive switching mechanisms, remains unclear. One proposal suggests that the current gradually increases as the filament forms and grows through the perovskite.^38^

Recently, a similar behavior observed in perovskite solar cells under inverse bias, commonly referred to as “breakdown”, has received increasing interest because of its relevance for device stability in a module.^39–41^ On the one hand, the phenomenon has been assigned to filament formation^42^ while on the other hand, it has been attributed to paired electrochemical reactions between the perovskite and the electrodes.^43^ In both cases, the relationship between the observed current change and the underlying mechanism remains unclear.

Here, we demonstrate that the capacitive hysteresis, the inductive hysteresis, the abrupt switching, and even a hysteresis-free response can be observed within the same perovskite-based device under varying conditions. Using in situ, real-time optical microscopy, we directly observe the morphological changes corresponding to transitions between these behaviors, particularly focusing on the formation and evolution of the conductive filament. We employ electron microscopy, conductive atomic force microscopy (c-AFM), and energy-dispersive X-ray spectroscopy (EDX) to further understand the mechanisms behind these resistance dynamics, especially their relationship to the filament dynamics. Moreover, we highlight the limitations of cyclic voltammetry as a standalone technique for distinguishing the mechanisms behind the different hysteresis mechanisms and demonstrate the value of complementary methods such as transient voltage measurements and impedance spectroscopy for unambiguously distinguishing these responses and mechanisms. We observe similar behavior using gold contacts, demonstrating that these findings are relevant for a wide range of perovskite-based devices, including both memristors and solar cells.

To investigate resistance dynamics in perovskite-based devices, we begin with a simple structure comprising a MAPbI_3_ perovskite film with two silver contacts on top, as depicted in Figure 1a. Figure 1b to e illustrates how this device exhibits all four resistance dynamics discussed above, depending on the measurement conditions during cyclic voltammetry. Specifically, Figure 1b shows capacitive (or normal) hysteresis, observed when the fresh device is measured in the dark. Figure 1c demonstrates inductive (or inverted) hysteresis, when the fresh device is measured under illumination. If the device is kept under continuous illumination and 10 V DC bias, the inductive behavior transitions to a hysteresis-free response after approximately 10 min, as shown in Figure 1d. Further exposure to these conditions leads to an abrupt switching response, which emerges after an additional 62 min, as illustrated in Figure 1e. Figure S1 shows the same measurements on a logarithmic scale. All measurements were conducted in a nitrogen atmosphere to maintain device stability.

**Figure 1:**
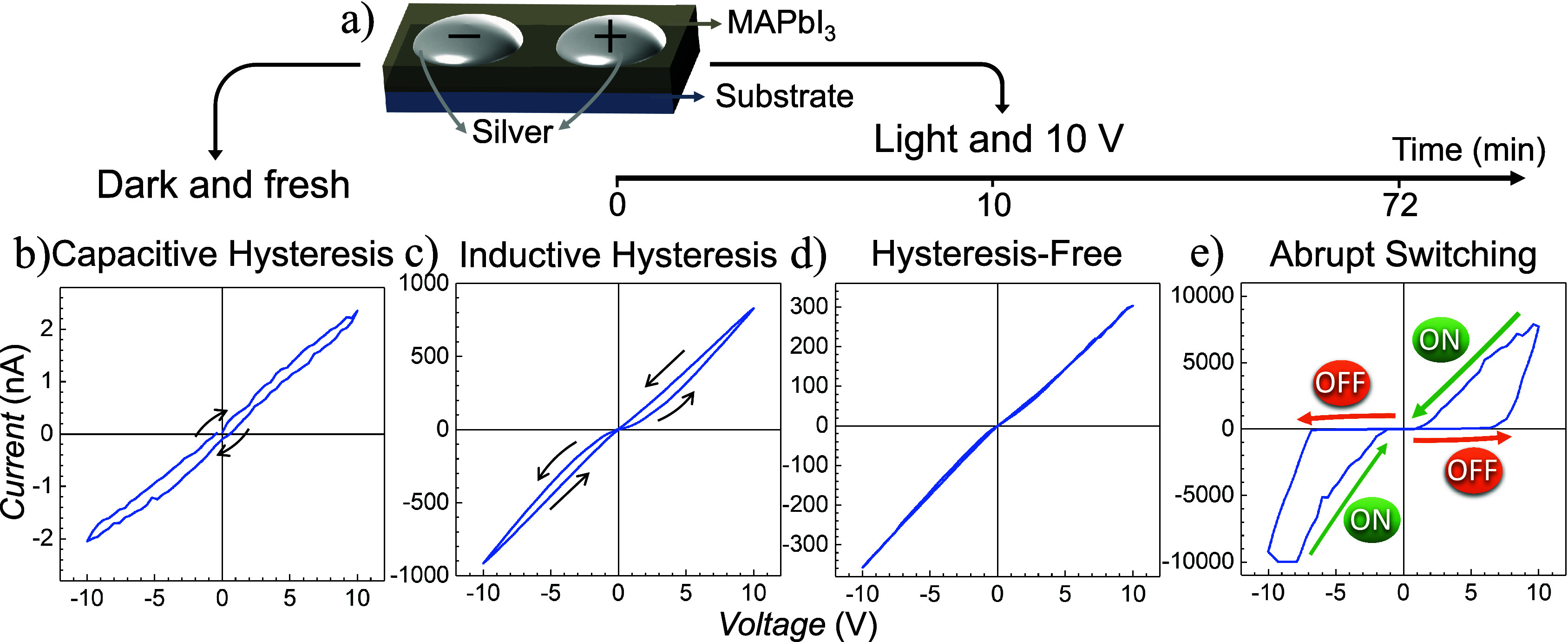
a) Schematic representation of the device structure, consisting of a MAPbI_3_ perovskite film with two silver contacts. b) Capacitive (or normal) hysteresis observed in a fresh device under dark conditions. c) Inductive (or inverted) hysteresis observed in a fresh device under illumination. d) Hysteresis-free response after approximately 10 min of continuous illumination and a 10 V bias. e) Emergence of abrupt switching response after 72 min of continuous illumination and 10 V bias.

To investigate the mechanisms behind the different resistance dynamics, we employed in situ, real-time optical microscopy while keeping the device under illumination and applied bias. These measurements, shown in Figure 2, were performed between the CV scans presented in Figure 1. The lateral configuration, with electrodes positioned with a gap of around 70 μm, enabled dynamic monitoring of the formation and growth of the filament.

**Figure 2:**
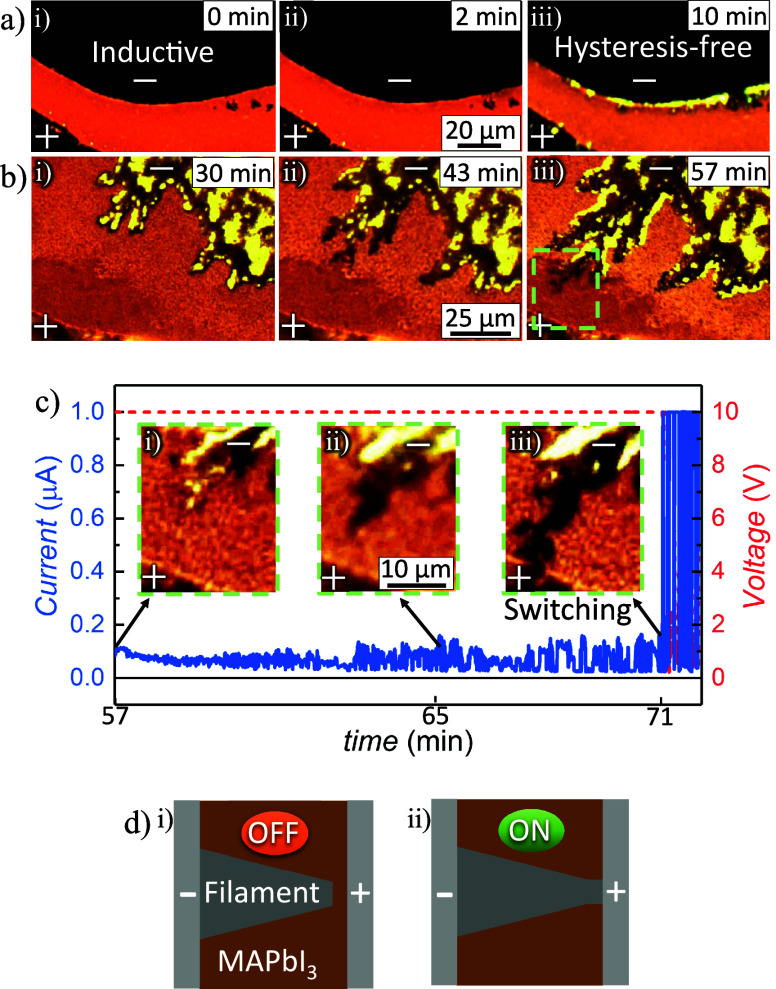
Photoluminescence (PL) microscope maps showing the evolution over time (minutes indicated on the top right) under applied illumination and bias (with the + and – indicating the positive and negative electrodes, respectively): a) the transition from (i) inductive behavior to (iii) hysteresis-free behavior; and b) the formation of a filament. c) Zoomed-in frames from panel b) capturing filament growth, with a sudden increase in current once the filament bridges the electrodes, as seen in (iii). d) Schematic illustrations of the high-resistance ″OFF″ state (filament disconnected) and the low-resistance ″ON″ state (filament connected), also observed in the CV in Figure 1e, highlighting the reversible switching between the two states.

The evolution of the morphological changes was recorded under a 10 V bias, with illumination provided by a blue (450 nm) LED from the back of the device. To filter out the excitation light, we placed a 650 nm long-pass filter between the sample and the camera, blocking blue light and capturing only the photoluminescence (PL) emission from the perovskite. As shown in red in Figure S2a (with a zoomed view in Figure S2b), the perovskite film exhibits a strong PL emission above 650 nm, peaking around 770 nm, which passes through the filter and appears orange/red in the camera image. In contrast, the signal from the silver electrode, shown in blue in Figure S2a and b, is negligible, resulting in dark regions in the camera image, except for possible reflections. This setup enables a clear visual distinction between the bright orange/red PL of the perovskite and the dark regions corresponding to the silver.

The continuous morphological changes can be seen in Videos S1, S2, and S3 in the Supporting Information. Video S1 captures the initial morphological changes, corresponding to the transition from inductive to hysteresis-free behavior, Video S2 shows the filament growth, while Video S3 focuses on the filament responsible for the abrupt switching behavior. Video S1 was recorded in a preliminary region, whereas Videos S2 and S3 were captured in the primary area (shown in dark green in Figure S2b, with a magnified view provided in Figure S2c) where filament growth was most pronounced.

Figure 2a presents three frames from Video S1. The first frame (Figure 2a-i) shows the fresh device under illumination, exhibiting inductive behavior, also referred to as inverted hysteresis, as shown in Figure 1c. After 2 min (Figure 2a-ii), no significant changes are observed. However, by the 10th minute (Figure 2a-iii), clear morphological changes appear at the interface between the perovskite layer and the negative silver electrode. This transition corresponds to the disappearance of the inductive behavior, resulting in a hysteresis-free response, as shown in Figure 1d. These observations establish a direct correlation between the disappearance of inductive behavior and morphological changes at the perovskite-electrode interface. These in situ observations directly link interfacial morphology to the inductive behavior. This finding supports previous studies suggesting that the mechanism behind inverted hysteresis and negative capacitance (or inductive behavior) is interfacial in nature, as modifications at the interface have been shown to suppress this process.^2,26,44^

Figure 2b provides three frames from Video S2, revealing that these interface changes evolve into filament formation. Filaments formation in perovskite devices with silver contacts have been previously reported.^38^ We intentionally employed silver electrodes to facilitate this process, as silver filaments are known to influence resistance dynamics. Figure S2c and d shows the filament illuminated with white light from above. Throughout the filament growth stages, the device exhibits a hysteresis-free response, as in Figure 1d, with no evidence of abrupt switching behavior. This finding confirms that the abrupt switching behavior, depicted in Figure 1e, does not yet appear during filament growth.

Figure 2c presents three zoomed-in frames from Video S3, focusing on the light-green highlighted region in Figure 2b-iii, alongside the corresponding current and voltage traces over time. As the filament grows, the current remains relatively steady, although with substantial noise. Once the filament bridges the gap between electrodes, as seen in Figure 2c-iii, the current abruptly rises to the set compliance current of the measurement, and the voltage drops to account for the further reduction in resistance. This result demonstrates that abrupt switching occurs exactly when the filament connects the two electrodes, effectively creating a short circuit.

The abrupt resistance switching observed in Figure 2c corresponds to the abrupt switching shown in the CV of Figure 1e, also illustrated in Figure 2d with the schemes of the ″OFF″ and ″ON″ states. Initially, the device is in a high-resistance ″OFF″ state, where the filament does not contact the negative electrode. As the voltage increases, an abrupt transition to the low-resistance ″ON″ state occurs when the filament bridges the gap, creating a short circuit. Upon decreasing the voltage, the device remains in the ″ON″ state until approaching zero bias, where the resistance abruptly increases again, returning to the ″OFF″ state. This reversibility is evident from the electrical measurements, both the real-time measurements (Figure 2c) and the CV (Figure 1e). However, no corresponding change is visible in the PL mapping, as shown in Video S3, indicating that the bulk structure of the filament remains intact. This observation suggests that the disconnection occurs in a very localized region near the electrode-filament interface, below the optical resolution of our setup, where the contact of the filament with the negative electrode forms and dissolves during the transitions to the “ON” and “OFF” states, respectively. Additionally, the CV in Figure 1e shows that abrupt switching can also occur during reverse bias, transitioning to the “ON” state, and returning to the “OFF” state as the bias approaches zero.

We observe capacitive behavior in the dark, while the inductive, hysteresis-free, and abrupt switching behaviors were accessed under illumination. Although literature reports that all these resistive behaviors can occur both in the dark and under illumination,^26,28,34^ in our device, light was essential to reliably induce and monitor the full range of transitions, particularly to enable real-time observation of the associated morphological changes. Possibly, the faster ion migration under illumination means that these features might also be observable in the dark at much longer time scales.^15^ Therefore, we believe that the mechanistic insights established here are broadly applicable and can be generalized to other devices that exhibit similar behaviors, including memristors, which typically operate in the dark. Further investigation of these phenomena in different device architectures and under various conditions would be valuable for fully validating and extending our findings.

To gain deeper insight into the mechanism of filament formation, we conducted a detailed characterization of the filament, as shown in Figure 3. Figure 3a shows the same filament depicted in Figure 2c-iii, now visualized with electron microscopy. Figure 3a-i combines scanning electron microscopy (SEM, Figure 3a-ii) and energy dispersive X-ray spectroscopy (EDX), with individual element maps for Pb, Ag, and I displayed in Figure 3a-iii, 3a-iv, and 3a-v, respectively. These analyses confirm that the filament is primarily composed of silver, consistent with prior reports.^38^ Compared to optical microscopy, these images provide greater resolution, particularly around the filament-electrode contact region, responsible for the abrupt resistance switching. A detailed view of this interface region is provided in Figure S3, further highlighting the contact of the filament with the electrode.

**Figure 3:**
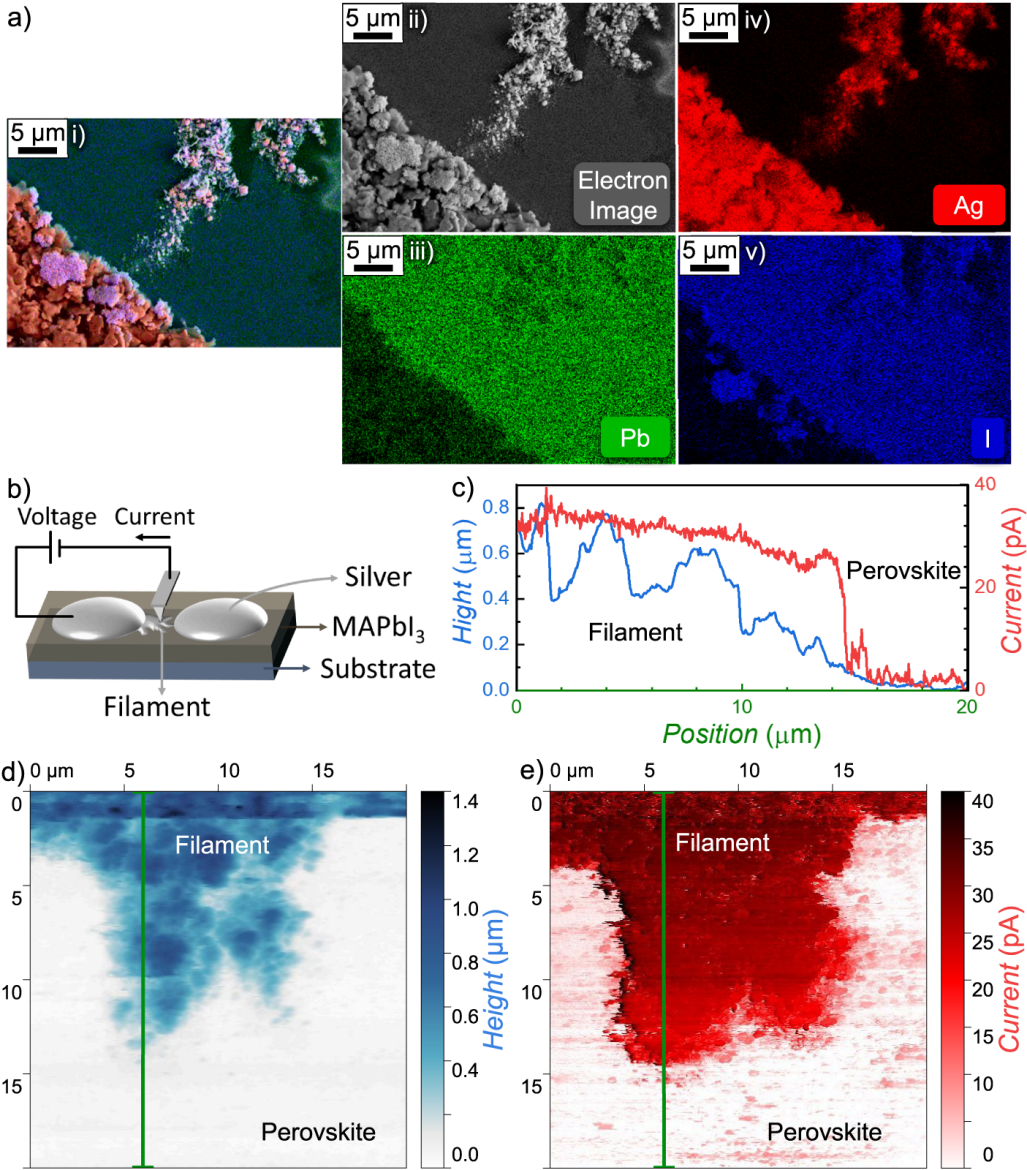
a) Electron microscopy characterization of the filament observed in Figure 2c. (i) Composite image combining SEM (ii) and EDX mappings for Pb (iii), Ag (iv), and I (v), confirming the filament is primarily composed of silver. b) Schematic of the conductive AFM (c-AFM) setup for simultaneous current and height mapping under bias. c) Line profile of height and current along the green lines in the height d) and current e) maps of the filament, demonstrating a strong correlation between elevated regions and high current.

To investigate the electrical properties of the filament, we employed conductive atomic force microscopy (c-AFM). The measurement setup is illustrated schematically in Figure 3b, where the height is mapped simultaneously with current measurements under a 300 mV bias using the AFM tip. The obtained height and current maps are shown in Figure 3d and 3e, respectively. Regions of high height align spatially with the areas of high current, as emphasized in Figure S4, where regions with height greater than 0.5 μm are highlighted in blue on the current map. This overlap is further analyzed in Figure 3c, which shows the extracted linear profiles of both height and current along the green lines indicated in Figure 3d and e (also Figure S4).

The height mapping reveals significant variations in filament morphology, with portions extending up to 0.8 μm above the surface—approximately double the thickness of the underlying perovskite layer. By contrast, the current map exhibits a step-like distribution, with near-zero current observed in areas with minimal height (perovskite film) and high current corresponding to regions of elevation (filament), although largely independent of precise height variations. Furthermore, as we move from the filament regions toward the perovskite layer, the height gradually decreases, approaching the filament tip. When the height becomes sufficiently low, the current drops sharply, indicating a transition from the low-resistance filament to the high-resistance perovskite film.

These results indicate that the filament is highly conductive. The local connection to the electrodes is the mechanism behind the observed abrupt switching behavior. This behavior is consistent with the sharp resistance change captured in Figure 2c when the filament bridges both electrodes, corresponding to the abrupt switching behavior previously shown in Figure 1e. The results also show little expansion of the conductive region beyond the morphological features of the filament, suggesting that little filament grows within the perovskite film.

CV is a widely used characterization technique that provides a straightforward analysis of optoelectronic devices, particularly perovskite devices. As shown in Figure 1, CV effectively identifies dominant resistive dynamics. However, as noted in the introduction, inductive and abrupt switching behaviors are often mistaken for one another in the literature, resulting in the assignment of different mechanisms to CV featuring similar responses.^34,38^ This confusion is understandable when comparing Figure 1c and e, because the current in the reverse scan (voltage decreasing) is higher than in the forward scan (voltage increase) in both measurements. However, as demonstrated in Figure 2 and Figure 3, the origin of these mechanisms is distinct. For instance, the diode-like behavior of the current seen in Figure 1c during the forward scan, followed by a linear decrease in current during the reverse scan, might be misattributed to filament formation. Yet, Figure 2 confirms the absence of optically resolvable filaments in the initial stages, and their appearance coincides with the disappearance of inductive behavior. We observe the inverted hysteresis from the very first CV measurement under illumination, which occurs on a time scale of less than a second. In principle, one or multiple small filaments could be responsible for this behavior, if they would form and dissolve extremely rapidly and remain undetectable by our PL imaging. However, we do not observe any such features with the other techniques employed, EDX or c-AFM, as shown in Figure 3.

Voltage transient measurements and impedance spectroscopy are powerful techniques for probing the underlying mechanisms in optoelectronic devices, particularly in perovskite-based systems.^45–47^ To provide a tool to differentiate the different hysteresis mechanisms clearly, we measured a lateral halide perovskite device with silver electrodes fabricated by UV-lithography using these techniques. The devices are similar to the devices above, but with more controlled dimensions of the electrodes, 10 mm long, separated by a gap of 50 μm. These large-area electrodes were designed to increase the current in the high-resistance state, thereby improving the accuracy of current measurements compared to the device used in Figure 2, where the active region is significantly smaller. The device exhibited the same four resistive dynamics observed in Figure 1 under comparable conditions, as shown in linear and logarithmic scales in Figure S5. Capacitive behavior (Figure S5a,e) was observed in the dark with the fresh device, while exposure to light initially induced an inductive behavior (Figure S5b,f). Prolonged light and bias exposure eventually suppressed the inductive feature, leaving a hysteresis-free response (Figure S5c,g). Finally, after additional light and voltage application, abrupt switching behavior emerged (Figure S5d,h). We performed these measurements at a scan rate of 1 V/s. As mentioned above, this is approximately the typical scan rate used in perovskite devices, allowing the observation of slow processes such as ionic migration. However, different scan rates can lead to varying hysteresis responses, as they can probe processes with different characteristic times. Transient voltage and impedance spectroscopy measurements were performed to investigate the underlying processes at each condition, as shown in Figure 4.

**Figure 4:**
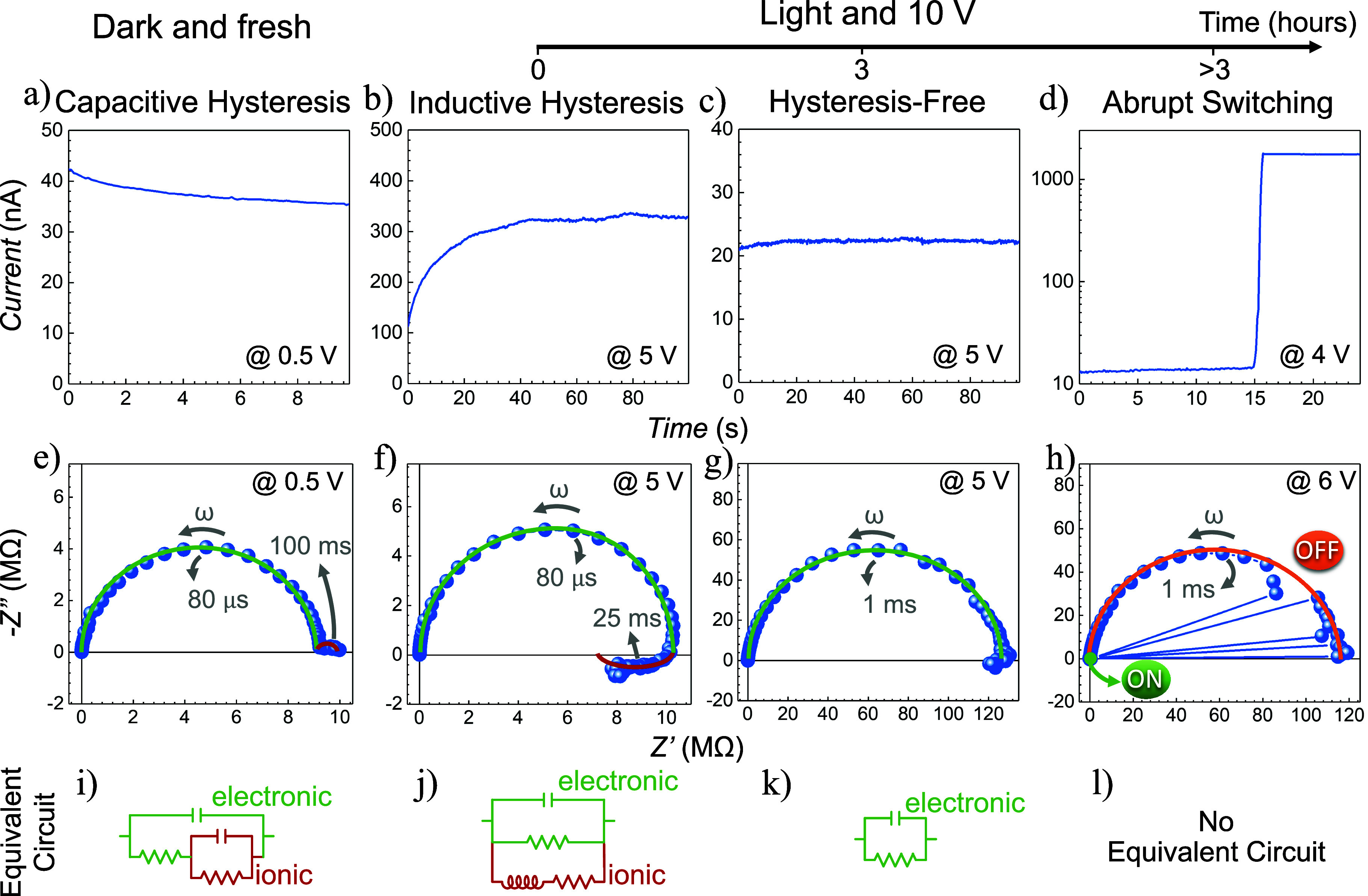
Transient current responses under a constant voltage for the device exhibiting the four resistive dynamics: a) capacitive, b) inductive, c) hysteresis-free, and d) abrupt switching behaviors. e-h) Corresponding impedance spectroscopy measurements for each behavior, highlighting distinct spectral features: the green arc represents an electronic process, while the red arc corresponds to an ionic process. The gray marks indicate the direction of increasing frequency and the characteristic times for each process. i-l) Equivalent circuit models used to interpret the impedance spectra. The green components represent a resistance-capacitance (RC) pair corresponding to the green arc in the spectra, while the red components represent either an RC pair for capacitive behavior or a resistance-inductor (RL) pair for inductive behavior.

Figure 4a-d show current responses for voltage transient, corresponding to the application of a constant positive voltage, indicated in each resistive dynamic figure. Prior to the bias application, the device was at equilibrium under zero voltage. Figure 4a (in the dark) shows a decaying current response, while Figure 4b (under light bias) shows a rising current, both consistent with capacitive and inductive behaviors, respectively. The current exhibits an exponential-like transient that stabilizes over time in both cases. Hysteresis-free behavior (Figure 4c, after 3 h 10 V bias application under light) produces a relatively stable current, while abrupt switching behavior (Figure 4d, longer than 3 h 10 V bias application under light) shows a sudden increase in current, corresponding to the filament-induced short circuit. The transient responses in Figure 4b and d clearly distinguish inductive and abrupt switching behaviors, a differentiation that can be difficult to make using cyclic voltammetry (CV) alone.

Figure 4e, f, g, and h show the impedance spectroscopy responses for the device exhibiting the different resistance dynamics. Correspondingly, Figure 4i, j, k, and l present the equivalent circuit models that can be used to analyze these spectra.

In Figure 4e, we observe the typical impedance response of a perovskite-based device, composed of two semicircles in the first quadrant, corresponding to capacitive responses. These responses can be modeled using two pairs of resistances and capacitors (RC circuits), as shown in Figure 4i. The characteristic time of each process can be determined by taking the inverse of the frequency at which the semicircle reaches its peak. The fast process (80 μs), highlighted in green, is associated with electronic processes, whereas the slower process (100 ms), shown in red, is linked to ionic processes.^13,48–51^ Given that the CV and transient voltage measurements operate on time scales of seconds, the electronic process is too rapid to affect these measurements significantly. Therefore, the differences observed in CV and transient voltage responses are attributed to the slower ionic processes. Specifically, the ionic process in Figure 4e (red arc) corresponds to a capacitive response, a feature extensively studied in perovskite solar cells, and associated with the normal (or capacitive) hysteresis observed in CV measurements, attributed to ion accumulation at interfaces.^12,25^

In Figure 4f, we see the impedance spectra for the device exhibiting inductive behavior. At high frequencies, there is an arc highlighted in green, similar to the one in Figure 4e, corresponding to an electronic process that remains too fast to impact CV or transient voltage measurements significantly. However, at low frequencies, we observe the emergence of an arc in the fourth quadrant, known as negative capacitance or inductance, which can be modeled using the circuit shown in Figure 4j. This feature has been observed in numerous perovskite-based devices and other technologies like dye-sensitized solar cells.^52,53^ More recently, it has been associated with inverted (or inductive) hysteresis in CV measurements, attributed to extra recombination caused by ion migration in perovskite solar cells.^2,26,54^ This behavior has also been leveraged in perovskite-based memristors,^55^ with studies confirming these observations through drift-diffusion simulations.^56,57^

Figure 4g presents the impedance response of a device exhibiting hysteresis-free behavior, achieved by applying light and bias until the inductive feature disappears. In this spectrum, the arc in the fourth quadrant (red) vanishes or becomes very small, leaving only the high-frequency electronic arc (green), which can be modeled simply with a resistor and a capacitor, as shown in Figure 4k.

Finally, Figure 4h shows the impedance response of the device exhibiting abrupt switching behavior. In this case, the impedance spectrum shows mainly a high-frequency arc, similar to Figure 4g, but with a unique feature: the impedance suddenly approaches zero at some frequencies. This sudden drop aligns with abrupt switching behavior, where resistance suddenly drops, corresponding to a significant drop in the real part of the impedance. The spectrum suggests that the impedance response matches a hysteresis-free behavior but with rapid transitions between low and high resistances. This resistor-like abrupt switching dynamic is a notable characteristic that demonstrates the abrupt switching response captured in our measurements.

In summary, our results demonstrate a clear progression of resistive dynamics: from capacitive (ion accumulation at interfaces), to inductive (additional recombination via ion migration), to hysteresis-free (no ionic contribution to the electrical response), and finally to abrupt switching (filament formation). These transitions are reflected in the CV, voltage transient, and impedance responses and are captured by evolving equivalent circuit models, from simple RC elements for capacitive behavior to circuits incorporating inductive branches to reproduce the inductive behavior. The choice of model elements directly corresponds to the dominant physical processes, with capacitive arcs representing ionic accumulation^13,48–51^ and inductive arcs indicating an extra recombination due to slow ionic migration.^2,26,54^ This systematic approach clarifies the mechanistic origins of each regime and provides a robust framework for interpreting similar behaviors in other perovskite-based devices.

Up to this point, our results have focused on perovskite devices with silver contacts. To demonstrate the generality of our findings, Figure 5 presents similar responses from devices with gold electrodes, shown in Figure 5a and b. Figure 5c–f confirms that these gold-contact devices exhibit the same four hysteresis behaviors in CV as the silver-contact devices in Figure 1. Additionally, Figure S6 presents these CV responses with the current on a logarithmic scale for clarity. Likewise, Figure 5g–j display the corresponding transient voltage responses for the four resistance dynamics, aligning with the trends observed in Figure 4a–d.

**Figure 5:**
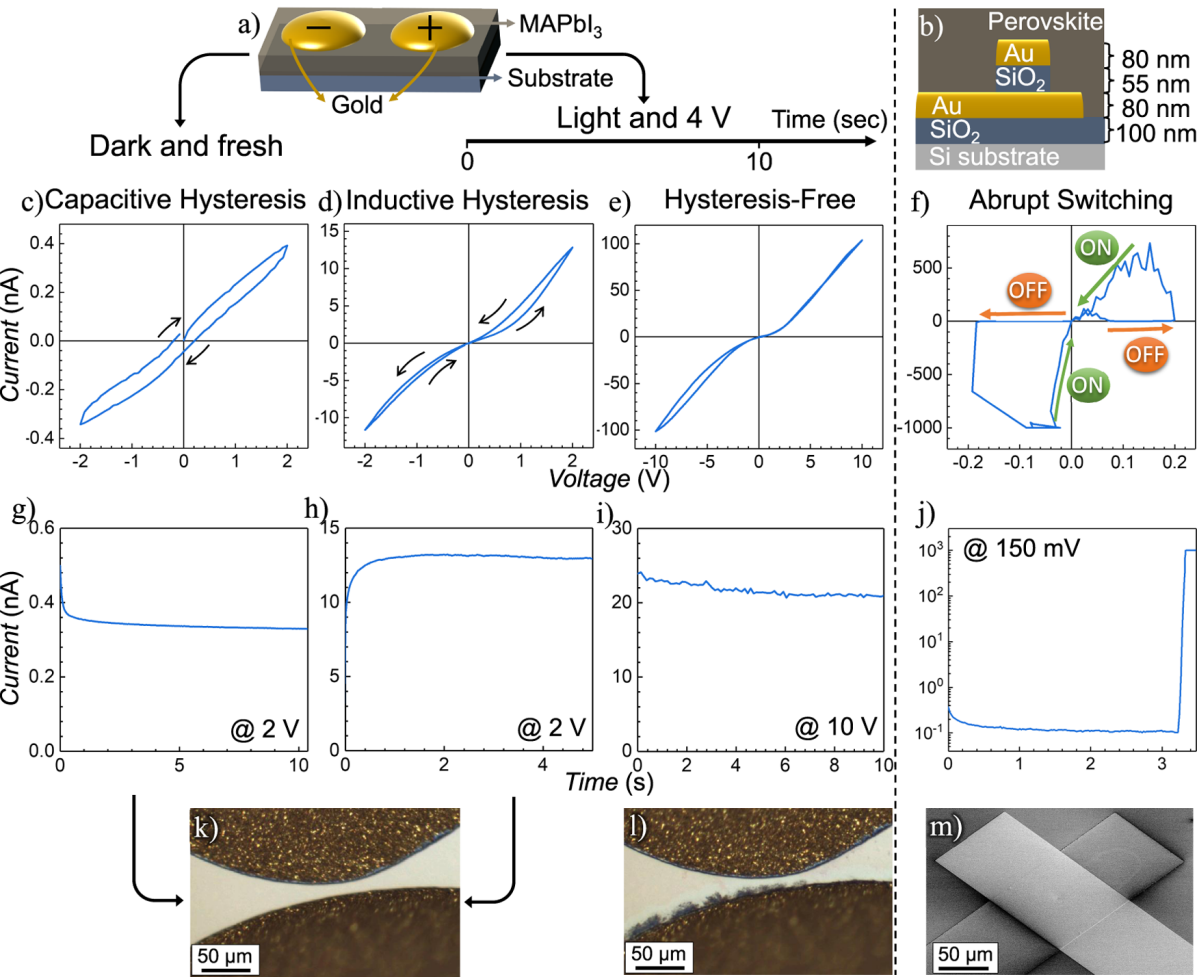
Gold-contacted perovskite devices under various conditions. a) Device configuration equivalent to that in Figure 1a but with gold contacts. b) Alternative configuration where the gold contacts form crossbars with a thin insulator in between.^5^ CV responses showing capacitive c), inductive d), and hysteresis-free e) behaviors for the device in a), and abrupt switching f) behavior for the device in b). g)-j) Transient voltage responses corresponding to c)-f), showing current decay g), current rise h), approximately stable current (i), and abrupt switching current with capacitive decay at short times j). Microscope images of the device in a) before k) and after l) prolonged exposure to light and bias, showing visible interface changes but no filament formation. m) SEM image of the device in b) before perovskite deposition.

Figure 5a shows a scheme of a device equivalent to that in Figure 1a with gold contacts instead of silver. Figure 5c-e displays CV responses for this gold-contact device under various conditions, exhibiting capacitive behavior in the dark (Figure 5c) and inductive behavior under illumination (Figure 5d), similar to the silver-contact device responses (Figure 1b and c, respectively). With prolonged exposure to light and bias, the inductive behavior disappears, and the device shows a hysteresis-free behavior at positive voltages (Figure 5e), similar to the silver-contact device (Figure 1d). Notably, the gold-contact device in Figure 5a does not exhibit abrupt switching behavior even after extended light and bias exposure (over 85 min at 10 V, 30 min at 16 V, and another 30 min at 21 V). Figure 5g–i illustrate transient voltage responses corresponding to these behaviors, similar to those observed for the silver-contact device in Figure 4a–c. Figure 5g and h shows the current decay and rise corresponding to capacitive and inductive behaviors, respectively, while Figure 5i indicates an approximately stable response (hysteresis-free). Figure 5k and 5l present microscope images of the gold-contact device in Figure 5a, before (Figure 5k) and after (Figure 5l) prolonged light and bias exposure. These images reveal significant interface changes that coincide with the disappearance of inductive behavior, leaving a hysteresis-free behavior, similar to observations in the silver-contact device (Figure 1 and Figure 2).

To clarify the dark features seen in Figure 5l, we performed EDX analysis shown in Figure S7. We observe morphological changes at the Au electrode-perovskite interface, producing dark features in optical and SEM images. EDX spectra reveal no accumulation of Au, Pb, or I in these regions; the perovskite composition remains homogeneous, and Au is only detected at the electrodes. Thus, no clear evidence of Au filament formation or halide vacancy clustering is observed. This behavior aligns with the literature reporting slower Au+ migration compared to Ag+, explaining the absence of abrupt switching in Au-contact devices.^58^ While small or unstable filaments cannot be fully excluded, they could be below our detection limits. The morphological changes and loss of inverted hysteresis support an interfacial ionic migration origin.^26^

On the other hand, Figure 5b presents a different configuration of a gold-contact device used in our recent study.^5^ In this device, the gold contacts form crossbars with a thin insulator between them, so that the perovskite remains in contact with both electrodes but with a much smaller gap of only 55 nm. This device exhibits abrupt switching behavior, as demonstrated by the CV response in Figure 5f, as previously reported in our earlier work,^5^ and by the transient current after three seconds of constant bias in Figure 5j. These responses closely resemble those of the silver-contact device, as shown in Figure 1e and Figure 4d, respectively. In Figure 5j, we notice a capacitive decay at shorter times, highlighting that multiple mechanisms can coexist under certain conditions. Finally, Figure 5m shows an SEM image of the device in Figure 5b before perovskite deposition. The small separation between the gold electrodes, along with complete perovskite coverage, prevents direct observation of filament formation during operation. However, the similarity of the abrupt switching response suggests that filament formation likely occurs in the gold device as well, although only at much smaller electrode separations.

These observations are consistent with the higher reactivity known for silver compared to gold:^58–60^ silver readily forms stable and extended filaments, while gold, being less reactive, would be expected to form smaller or less stable filaments, requiring shorter distances to bridge contacts. Alternatively, if the filaments arise from vacancy migration within the perovskite as has been proposed in some studies,^37,61^ such filaments are also expected to require shorter distances than silver filaments.

To summarize, we identified and characterized four distinct hysteresis behaviors in halide perovskite devices: capacitive, inductive, hysteresis-free, and abrupt switching. All four responses can be observed in a simple Ag/perovskite/Ag device. Capacitive and inductive hysteresis appear in fresh devices under dark and illuminated conditions, respectively. Using cyclic voltammetry and in situ real-time photoluminescence microscopy, we show that continuous bias and light modify the perovskite–electrode interface, leading to the disappearance of inductive hysteresis and the emergence of a hysteresis-free response, supporting the interfacial origin of inductive behavior. Further bias and illumination promote the formation of conductive filaments. Abrupt resistance switching occurs only when a filament bridges both electrodes, forming a short circuit. This switching is fully reversible, with the bulk filament remaining intact; the resistance state change arises from reversible contact at the filament–electrode interface. Conductive AFM and electron microscopy confirm that the filaments are highly conductive and composed of silver. Voltage transients and impedance spectroscopy are shown to be effective tools for distinguishing the hysteresis modes. Similar behaviors are also observed in gold-contact devices, though abrupt switching only occurs at nanometer-scale separations, suggesting filament formation still takes place, possibly of gold or vacancies, but with smaller and less stable filaments due to the lower reactivity of gold.

## Supplementary Material









## Notes

Experimental details, including materials, device fabrication protocols, and measurement methods; additional data and analyses comprising cyclic voltammetry (linear and logarithmic scales); in situ and ex-situ optical microscopy, photoluminescence, electron microscopy (SEM/EDX), and atomic force microscopy characterizations (PDF)
Video S1 (MOV)
Video S2 (MOV)
Video S3 (MOV)
