# Supplementary material for: Hysteresis in Perovskite Devices: Understanding the Abrupt Resistive Switching Mechanism
Source: ACS Energy Lett. 2025 Jul 24;10(8):3983–92. doi: 10.1021/acsenergylett.5c01556 (PMC12340948; doi:10.1021/acsenergylett.5c01556)
Supplement: Supplementary file 1 [file nz5c01556_si_001.pdf]

Supporting information

Hysteresis in Perovskite Devices: Understanding the Abrupt Resistive Switching Mechanism

Agustin O. Alvarez,[a]* *Jeroen J. de Boer,*[a] *Lars Sonneveld,*[a] *Yorick Bleiji,*[a] *Esther Alarcón-Lladó,*[a] *and Bruno Ehrler*[a]*

LMPV-Sustainable Energy Materials Department, AMOLF, Science Park 104, 1098 XG Amsterdam, The Netherlands

E-mail: a.alvarez@amolf.nl; b.ehrler@amolf.nl

Experimental Section

Device Fabrications

Quartz and p-doped Si wafers were purchased from Siegert Wafer. PbI_2 (99.99%) was purchased from TCI. Methylammonium iodide (MAI) was purchased from Solaronix. Anhydrous DMF and chlorobenzene were purchased from Sigma-Aldrich. 950 PMMA A8 was purchased from Kayaku Advanced Materials. All materials were used without further purification.

Crosspoint devices were fabricated using a previously described procedure[1]. Briefly, gold bottom electrodes were patterned on the Si wafer with a 100 nm thermal oxide layer with a lift-off procedure using MA-N1410 resist. The resist was UV-exposed on a Süss MA6/BA6 mask aligner and developed in MA-D533/s. A 5 nm Cr adhesion layer and an 80 nm Au layer were deposited on the resist by e-beam physical vapor deposition. The wafer was then soaked in acetone for one hour for lift-off. A 60 nm SiO_2 insulating spacer layer was deposited by inductively coupled plasma-enhanced chemical vapor deposition in an Oxford PlasmaPro100 ICPCVD system from an O_2 and SiH_4 gas mixture. The gold top contacts were patterned using the same procedure as the bottom contacts. The SiO_2 spacer layer was plasma-etched off the bottom electrodes with an Ar and CHF_3 gas mixture in an Oxford Plasmalab 80 Plus system. The top electrodes were used as a hard-mask in this process.

The lateral electrodes were patterned on quartz wafers using the same lift-off process described above, but instead of 80 nm of gold, 80 nm of silver was deposited on the patterned resist.

The halide perovskite layers were prepared inside a nitrogen-filled glovebox (< 0.5 ppm O_2 and water). First, a 40 wt% $MAPbI_3$ precursor was prepared by dissolving a stoichiometric mixture of PbI_2 and MAI in DMF. The precursor was spin-coated on crosspoint and lateral electrode chips at 4000 rpm for 30 seconds. Chlorobenzene was added after 3 seconds of spinning. The chips were annealed at 100 °C for 10 minutes immediately after spin-coating. Finally, a PMMA capping layer was spin-coated on the chips from the 950 PMMA A8 solution at 3000 rpm for 45 seconds, followed by a 5-minute bake at 100 °C.

PELCO® Conductive Silver Paint and PELCO® Conductive Gold Paste were used to deposit the respective electrodes onto the perovskite. The pastes were chosen to ensure sufficient silver/gold availability while facilitating reliable wire attachment. The compact active area, with electrodes positioned close together in a small region, allowed precise control over potential filament formation during microscopy observations.

Electrical Characterization

Fluxim's Paios system was employed to measure cyclic voltammetry, transient voltage, and impedance spectroscopy on the silver contacts with a parallel configuration. The standard white LED at 100% from the Paios served as the light source. For the impedance spectroscopy, a perturbation of 200 mV was applied, and the frequency was swept between 10 MHz and 1 Hz. All the rest of the electrical measurements were acquired using an Agilent B2902A instrument.

Optical Microscopy Characterization

The in-situ morphology evolution was captured using a WITec alpha300 SR confocal imaging microscope. A 450 nm LED was used as the light source to illuminate the sample. Behind the sample, we had the option of placing a 650 nm long-pass filter to remove the light from the LED transmitted through the sample. Then, the detector behind the filter receives only the photoluminescence light from the sample. For photoluminescence spectral measurements, the LED was replaced by coupling a Thorlabs S1FC405 405 nm continuous-wave diode laser directly to the WITec microscope as the excitation source.

Electron Beam Characterization

SEM and Energy Dispersive X-ray spectroscopy (EDX) measurements were performed on a FEI Verios 460L scanning electron microscope with an Oxford Instruments X-Max 80 EDX detector. An accelerating voltage of 15 kV with a beam current of 200 pA was used for an acquisition time of 10 minutes. The sample was mounted on a sample holder using copper clamps, ensuring electrical grounding of the silver electrodes to the sample stage. The sample was measured uncoated. Oxford AZtec was used for data analysis.

Atomic Force Microscopy Characterization

Topographical and current maps were obtained with atomic force microscopy (AFM), using a Bruker Dimensional Icon and a solid Pt tip (Rocky Mountain Nanotechnology, model 25PT400B, nominal tip radius < 8 nm). The AFM was placed inside a fume hood while using a flow of nitrogen to minimize the humidity close to the sample. The sample was measured in an enclosure to enable conductance measurements in the dark. The height and current data were measured simultaneously in contact mode while applying a bias of 300 mV between the tip and the silver electrode on the perovskite device. The size of the maps was 20 µm, 512 by 512 pixels, and a scan rate of 0.3 Hz was used. The AFM data were treated using the software Gwyddion (version 2.65).

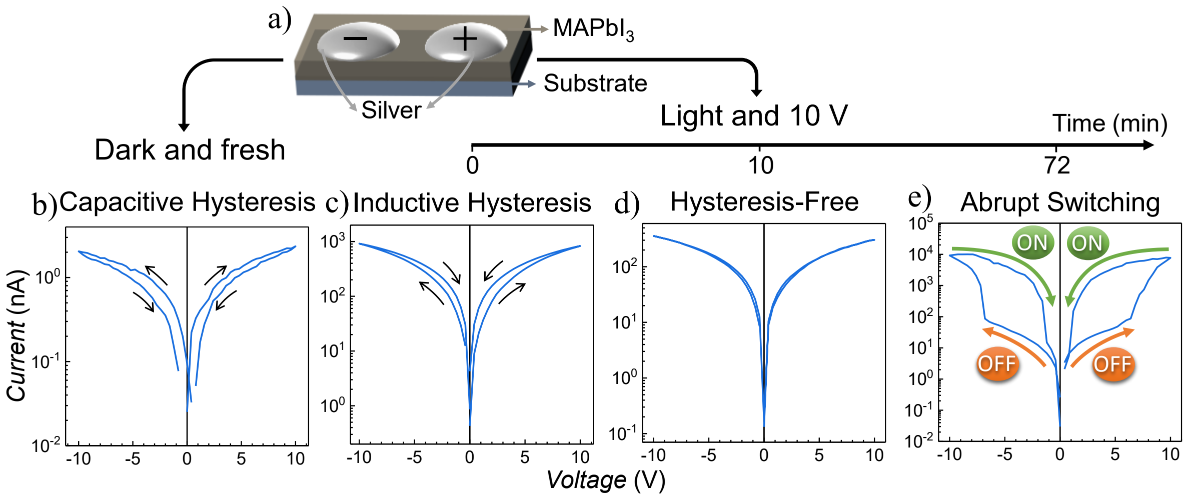

Figure S1. a) Schematic representation of the device structure, consisting of a MAPbI3 perovskite film with two silver contacts. b) Capacitive (or normal) hysteresis observed in a fresh device under dark conditions. c) Inductive (or inverted) hysteresis observed in a fresh device under illumination. d) Hysteresis-free response after approximately 10 minutes of continuous illumination and a 10 V bias. e) Emergence of abrupt switching response after 72 minutes of continuous illumination and 10 V bias. Current is represented on a logarithmic scale.

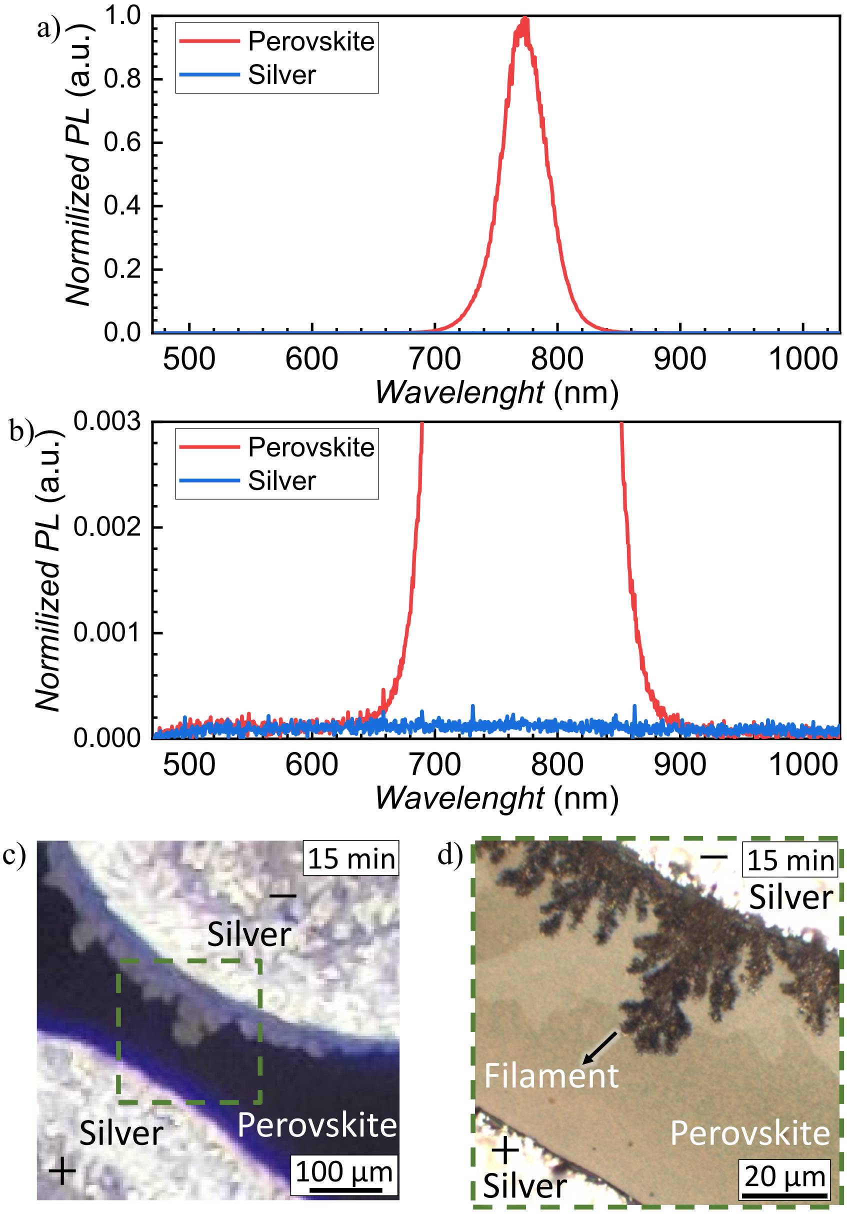

Figure S2. a) Normalized photoluminescence (PL) spectra of the perovskite film (red) and silver electrode (blue) measured with a Thorlabs S1FC405 405 nm continuous-wave diode laser. (b) Zoomed view showing strong perovskite emission above 650 nm and negligible PL from silver, which enables clear contrast between bright orange/red perovskite and dark silver regions in the PL mapping shown in **Figure 2**. c) Optical microscope image of the perovskite device under white light illumination, with the region of interest highlighted by a green dashed square. d) Higher-magnification image of the area marked in c), showing the filament illuminated from above.

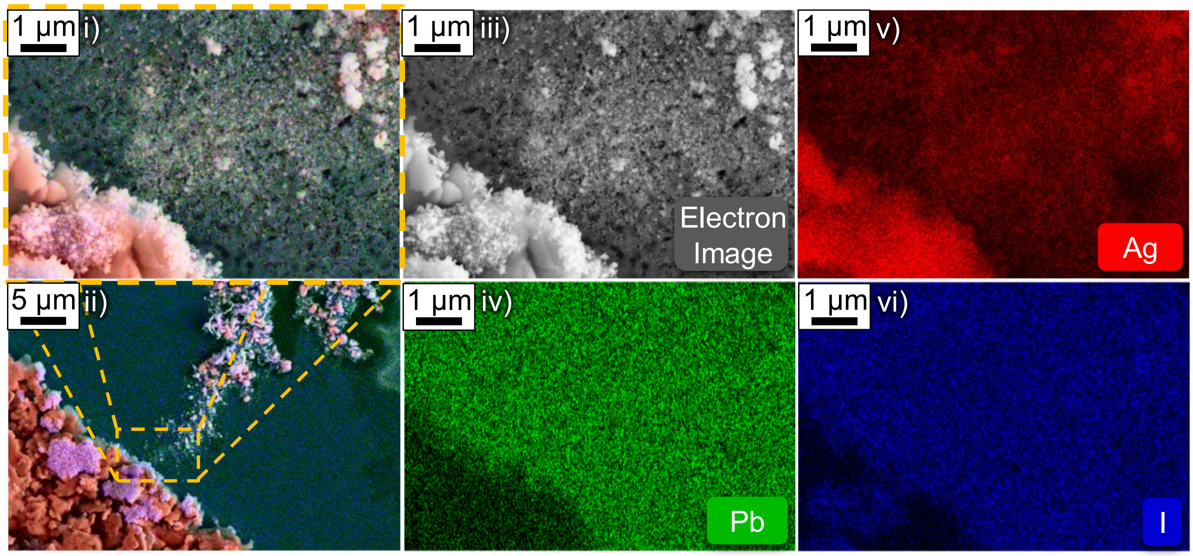

Figure S3. Electron microscopy characterization of the filament, zoomed-in from **Figure 3**a of the main text, from the region marked in ii). i) Composite image combining SEM iii) and EDX mappings for Pb iv), Ag v), and I vi), emphasizing the continuity of the filament into the electrode region.

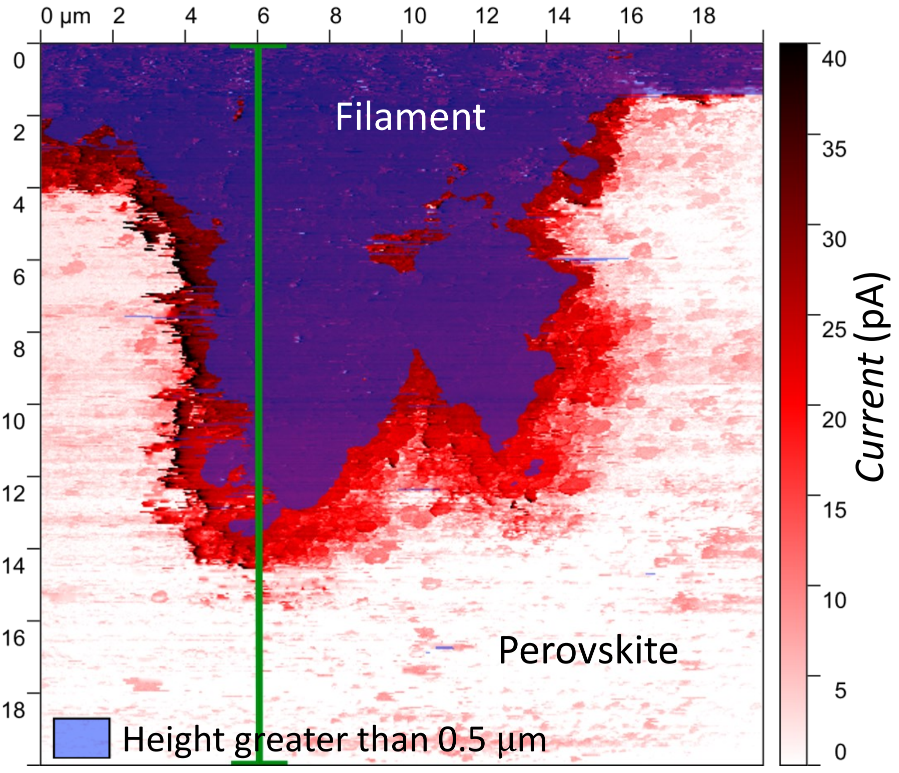

Figure S4. Overlay of current mapping (red) and height mapping (blue, regions above 0.5 µm) from c-AFM measurements, showing strong spatial correlation between high-current areas and elevated filament regions.

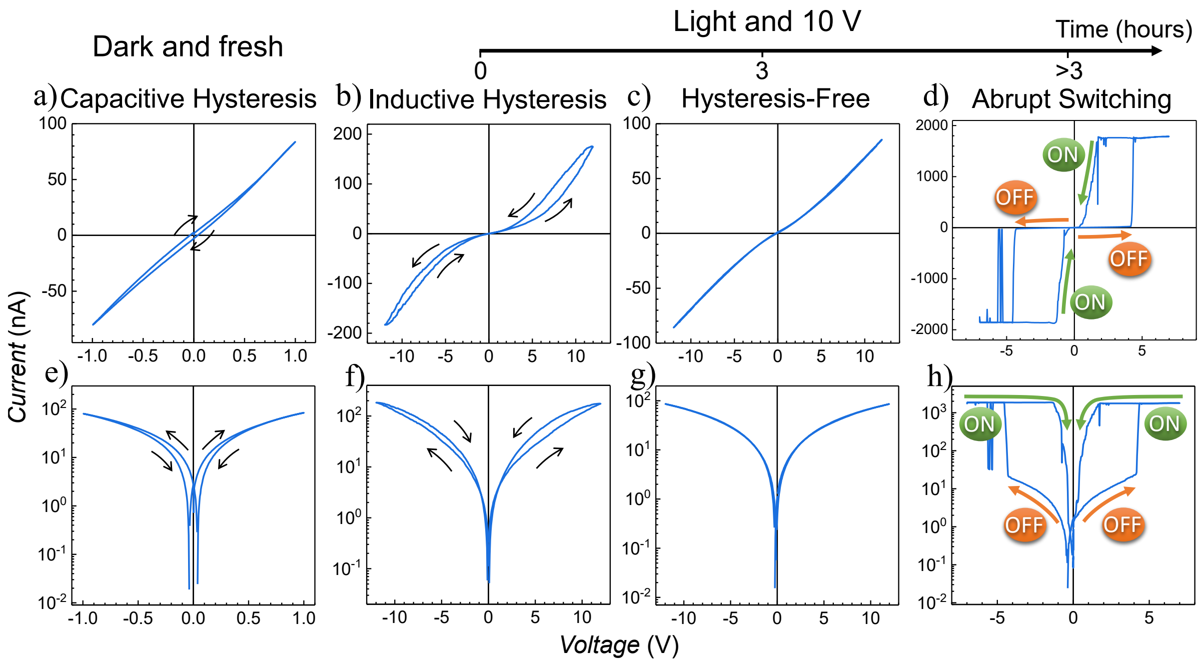

Figure S5. Cyclic voltammetry (CV) measurements for the device with silver electrodes in a channel geometry, presented in linear (a-d) and logarithmic (e-h) scales. a, e) Capacitive hysteresis observed in the dark with the fresh device. b, f) Inductive hysteresis emerging after initial light exposure. c, g) Hysteresis-free behavior resulting from prolonged light and voltage exposure. d, h) Abrupt switching behavior occurring after additional light and voltage application. These different behaviors correspond to the voltage transient and impedance spectroscopy responses shown in **Figure 4**.

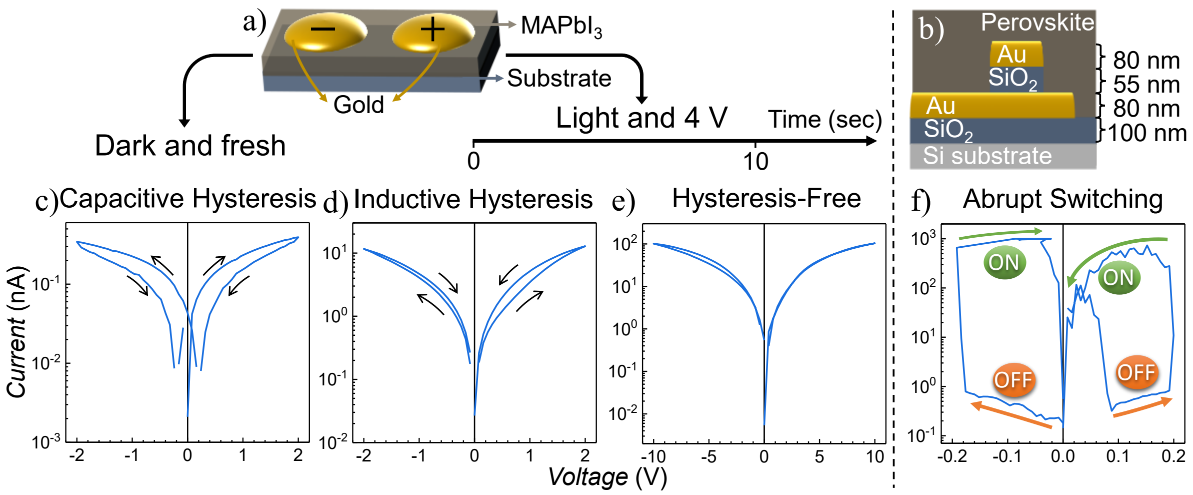

Figure S6. Gold-contacted perovskite devices under various conditions. a) Device configuration equivalent to that in **Figure 1**a but with gold contacts. b) Alternative configuration where the gold contacts form crossbars with a thin insulator in between[1]. CV responses, with current in logarithmic scale, showing capacitive c), inductive d), and hysteresis-free e) behaviors for the device in a), and abrupt switching f) behavior for the device in b).

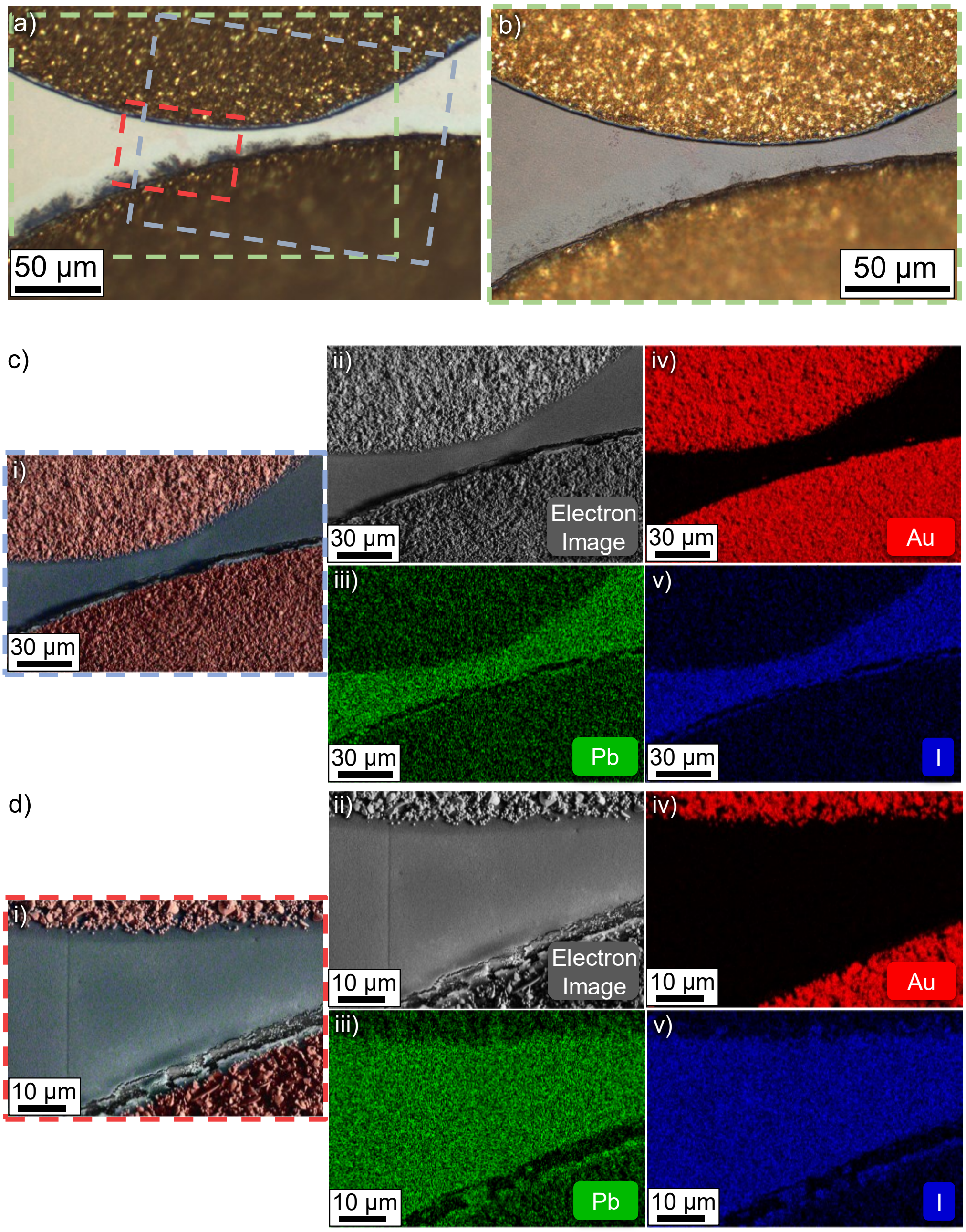

Figure S7. EDX analysis of the Au-contact device interface corresponding to Figure 5l. a) Optical image after prolonged light and bias exposure, with the green dashed square indicating the region re-imaged after nine months in b), showing less pronounced but still visible dark features. Blue and red squares mark EDX measurement areas shown in c) and d), each showing: i) combining SEM ii) and EDX mappings for Pb iii), Au iv), and I v). The maps show homogeneous Pb and I in the perovskite and Au confined to the electrode, with no evidence of Au accumulation or compositional anomalies that suggest the presence of filaments.

References

[1] J. J. de Boer, B. Ehrler, Scalable Microscale Artificial Synapses of Lead Halide Perovskite with Femtojoule Energy Consumption, *ACS Energy Lett.* **2024**, *9*, 5787-5794. https://doi.org/10.1021/acsenergylett.4c02360.
